# The Digital Divide Amongst High-Need High-Risk Veterans

**DOI:** 10.1093/geroni/igab046.2407

**Published:** 2021-12-17

**Authors:** Shirley Li, Kiranmayee Muralidhar, Fei Tang, Willy Marcos Valencia, Stuti Dang

**Affiliations:** 1 University of Miami Miller School of Medicine, Boynton Beach, Florida, United States; 2 University of Miami Miller School of Medicine, Miami, Florida, United States; 3 Miami Veteran Affairs Healthcare System, Miami, Florida, United States; 4 Medical University of South Carolina, Miami, Florida, United States; 5 Miami Veterans Affairs Healthcare System, Miami, Florida, United States

## Abstract

High-need high-risk (HNHR) veterans are medically complex and at the highest risk of hospitalization and long-term institutionalization. Technology can mitigate challenges these veterans have in accessing healthcare. Willingness to use technology as well as access and ability to use technology were assessed in this study. At the time of the survey, 2543 Miami VAHS veterans were listed as HNHR. 634 veterans ultimately completed the questionnaire, and 602 answered the “willingness to use video-visits” question. Of the 602 respondents, 327 (54.3%) reported they were willing for video-visits with the VA, while 275 (45.6%) were not. Those who were willing were significantly younger (P<0.001), with higher educational qualifications (P=0.002), and more health literate than those not willing (P<0.001). They were more also capable of using the Internet, more likely to use email and be enrolled in the VA’s patient portal, My HealtheVet (P<0.001). However, of the veterans who were willing, 248 (75.8%) had a device with video-capable technology. Those with video-capable technology were younger (P=0.004), more health literate (P=0.01), and less likely to be Black or African American (P=0.007). They were more capable of using the Internet, more likely to use email, and be enrolled in My HealtheVet than those without (P<0.001). Half of the respondents were willing for video-visits but a quarter of those willing lacked requisite technology, thereby making only about 41.2% of the respondents willing and video-capable. To minimize the digital divide, especially during the ongoing COVID-19 pandemic, targeted measures need to address these disparities in this vulnerable population.

